# The contribution of viral toxins to infection and pathogenesis

**DOI:** 10.1128/mbio.00421-25

**Published:** 2026-03-13

**Authors:** Scott B. Biering, Henry Puerta-Guardo, Felix Pahmeier, Vasiliya Kril, Eva Harris

**Affiliations:** 1 Department of Molecular Biology, School of Biological Sciences, University of California 8784, San Diego, La Jolla, California, USA; 2 Laboratorio de Virologia, CIR-Biomedicas y Unidad Colaborativa de Bioensayos Entomologicos (UCBE), Universidad Autonoma de Yucatan 27778, Merida, Mexico; 3 Division of Infectious Diseases and Vaccinology, School of Public Health, University of California, Berkeley 1438, Berkeley, California, USA; The Ohio State University, Columbus, Ohio, USA

**Keywords:** viral dissemination, vascular leak, dengue virus, SARS-CoV-2, Ebola virus, Crimean-Congo hemorrhagic fever virus, DENV NS1, SARS-CoV-2 Spike, EBOV GP, CCHFV GP38, viral pathogenesis

## Abstract

The process by which viruses cause disease, viral pathogenesis, is the result of both infection of cells and the host immune response. A less studied but equally important contributor to viral pathogenesis is viral dissemination, the capacity of a virus to move from the primary site of infection, traverse physiological barriers, and gain access to secondary sites of infection. This dictates viral tropism and pathogenesis, but the mechanisms governing barrier crossing are incompletely understood. While the presence of viral receptors on cells is a major determinant of viral tropism and a prerequisite for infection, it does not completely explain the capacity of viruses to enter a tissue. Our recent work has begun to characterize the contribution of soluble viral proteins, acting as “viral toxins,” to viral dissemination, tissue tropism, and overall pathogenesis within an infected host. In this review, we discuss the characteristics of these viral toxins, which are soluble or surface-exposed viral proteins that can interact with endothelial and/or epithelial barriers, as well as immune cells, to trigger signaling pathways, resulting in the transient breakdown of cellular structures maintaining barrier integrity. The disruption of these barriers induces vascular leak and facilitates virus dissemination, influencing viral tropism and pathogenesis. Importantly, blocking this process prevents leak, viral dissemination, and severe disease during infection, highlighting the value of therapeutic intervention against viral toxin activity. Here, we summarize our current understanding of recently discovered viral toxins from the *Flaviviridae, Coronaviridae, Nairoviridae,* and *Filoviridae*.

## INTRODUCTION

## OF VIRUSES AND BARRIERS

Viral pathogenesis is a complex process involving both the virus and the host. Tissue damage caused during viral infection can result from the virus and/or the host immune response. A prerequisite for host tissue damage is successful niche establishment by an invading viral pathogen. This requires the virus to enter a host, replicate at the primary site of infection, disseminate to and infect cells at both primary and secondary (distal) sites of infection, and finally, for some viruses, disseminate to sites of transmission to subsequent hosts. These stages can be broadly separated into cellular infection events and viral dissemination. Most virology research focuses on understanding the mechanisms of cellular infection. Indeed, cellular receptors required for viral entry into cells are major determinants of both viral and disease tropism, as well as the zoonotic host range of emerging viruses. Viral dissemination is also critical for niche establishment, pathogenesis, and viral transmission and directly impacts tissue and disease tropism; however, mechanisms of viral dissemination are less well studied.

**Fig 1:**
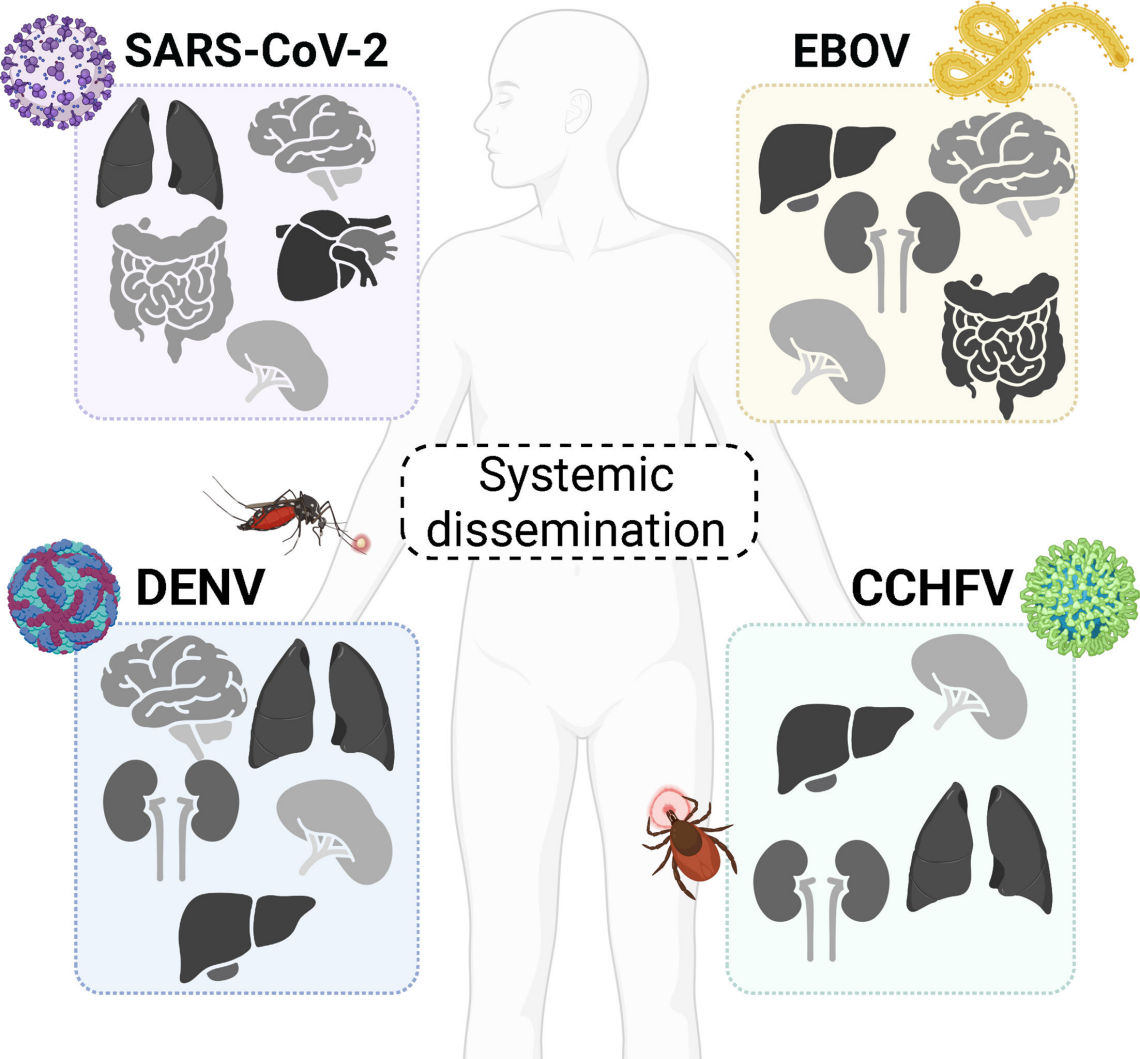
Systemic dissemination impacts tissue tropism of pathogenic viruses. Highly pathogenic air-borne viruses (e.g., SARS-CoV-2), bloodborne viruses (e.g., EBOV), or arboviruses (e.g., DENV, CCHFV) are able to disseminate systemically throughout the body and establish productive infection in multiple organs (e.g. lungs, brain, intestines, heart, kidneys, and spleen) impacting viral pathogenesis and disease outcome. DENV, dengue virus; CCHFV, Crimean-Congo hemorrhagic fever virus; SARS-CoV-2, Severe acute respiratory syndrome coronavirus 2; and EBOV, Ebola virus. Created in BioRender (Biering Lab, 2026, https://BioRender.com/4w5u3bc).

Viral infection can begin at multiple sites of the body, entering through mucosal surfaces including the respiratory, gastrointestinal, and genitourinary tracts or through the skin, such as after an insect bite in the case of arthropod-transmitted viruses (arboviruses) (Fig. 1). Viruses infect cells locally and can either disseminate to distal secondary sites of infection or remain at the primary site of infection (1–5). Before a virus can infect a cell, it must overcome multiple challenges, such as traversing physiological barriers present at the primary site of infection. These include extracellular matrix and collagen components in the dermis, mucins comprising the epithelial glycocalyx lining the lung and gut, and the endothelial glycocalyx, a network of glycoproteins and proteoglycans containing glycosaminoglycan side chains (6–8). Below the glycocalyx, both endothelial and epithelial cells are held tightly together by intercellular junctional complexes, including the tight and adherens junctions, thereby forming a physical barrier (9). After gaining access to and infecting cells at primary sites of infection, some viruses disseminate to secondary sites of infection, including the lymphatics, the endothelium, or the tissues of the liver, spleen, and, in some cases, the central nervous system (2, 9–12). While the details of target cell infection are a major area of research, the mechanisms viruses have evolved to disrupt and traverse physiological barriers are less well characterized but present an attractive therapeutic target.

## MICROBIAL STRATEGIES TO DISRUPT AND CROSS PHYSIOLOGICAL BARRIERS

**Fig 2:**
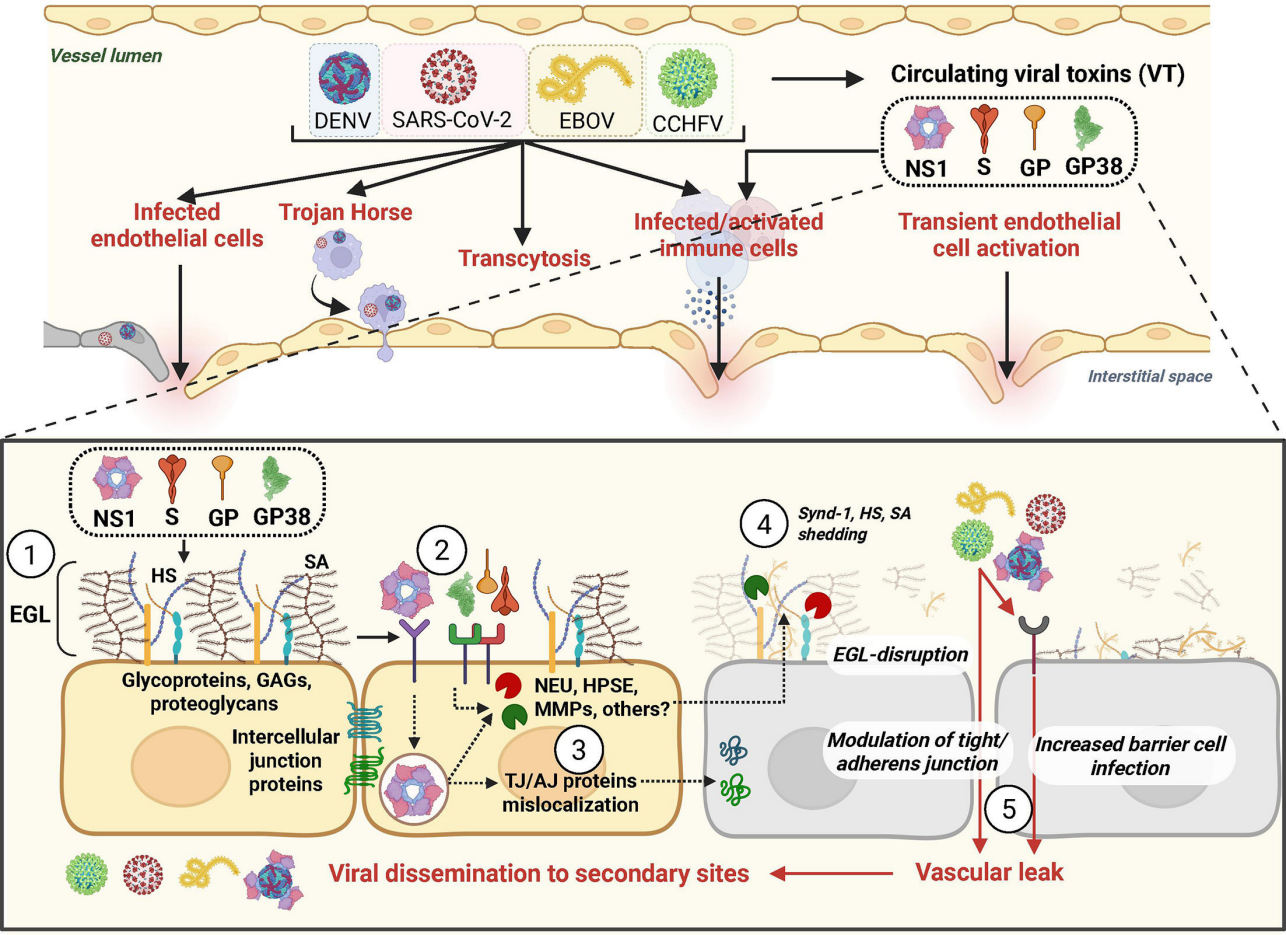
Viral toxin-mediated barrier dysfunction and other mechanisms of viral dissemination. Viruses use diverse strategies to cross cellular barriers. These comprise (from left to right) direct endothelial cell infection (lytic or non-lytic), infection of migrating immune cells crossing the endothelial barrier (Trojan horse strategy), and transcytosis through cellular barriers. Infected or activated immune cells can also indirectly contribute to virus dissemination through the secretion of proinflammatory cytokines detrimental to endothelial barrier integrity. Different viral families encode viral toxins, soluble proteins that trigger endothelial and/or epithelial barrier dysfunction and hyperpermeability. This mechanism involves interactions between viral toxins and barrier cells, with (1) DENV NS1, SARS-CoV-2 Spike, EBOV GP, and CCHFV GP38 interacting with the glycan components and (2) cellular receptors. (3) Following initial interactions, DENV NS1 is internalized into cells via clathrin-mediated endocytosis, whereas other viral toxins, such as Spike and GP38, remain at the cell surface. Signaling pathways are then triggered, leading to the activation of EGL-degrading enzymes such as neuraminidase (NEU), heparinase (HPSE), or matrix metalloproteinases (MMPs). In addition, viral toxins induce mislocalization of tight and adherens-junction proteins. (4) Finally, these pathways trigger shedding of EGL components, including Syndecan-1 (Synd-1), heparan sulfate (HS), and sialic acid (SA), (5) resulting in endothelial and epithelial barrier disruption and vascular leak, contributing to viral dissemination. Created in BioRender (Biering Lab, 2026, https://BioRender.com/4w5u3bc).

Several mechanisms have been reported to contribute to viral dissemination (Fig. 2). One common mechanism is the “Trojan horse” hypothesis wherein virus-infected immune cells disseminate across barriers, thereby providing the virus access to new tissues. This mechanism has been reported for multiple virus families, including the *Flaviviridae* and *Togaviridae* (2, 10, 13, 14). Transcytosis, or the active transport of viruses directly through a cell, independently of virus infection, is another mechanism utilized by viruses to cross barriers (10, 15, 16). Alternatively, infection of target cells, including epithelial and endothelial cells, can also result in passage across these barriers. This includes both lytic infection, common to viral hemorrhagic fever viruses (VHF), and non-lytic infection reported for some flaviviruses (10, 17). Thus, infection of target cells can result in direct breakdown of epithelial or endothelial barriers, promoting virus passage or simply the release of progeny virus across the barrier cell in the absence of cell death. In the case of arboviruses, mechanical damage resulting from the bite of an insect vector (probing) may also promote passage of viruses across barriers (18). General inflammatory pathways activated by viral infection can also promote virus crossing of barriers via several mechanisms. Tissue damage and cell death triggered by inflammatory responses can result in the breakdown of endothelial or epithelial barriers (19). Alternatively, inflammation can result in chemokine production that recruits highly permissive immune cell populations to the primary site of infection, as has been reported for murine norovirus (20). Similarly, for arboviruses, components of mosquito saliva mediate cutaneous recruitment of target myeloid cells, aiding arbovirus transmission (21–25). However, exactly how viruses hijack physiological processes to cross internal barriers and which viral proteins contribute to these processes are unclear.

More broadly, many microbes, both pathogens and commensals, have evolved diverse mechanisms to traverse or disrupt physiological barriers. These include bacteria directly encoding effectors or enzymes that can degrade protein or glycan components of the glycocalyx or trigger signaling pathways that modulate the integrity of the intercellular junctional complexes. Examples include the degradation of heparan sulfate via heparin lyases produced by *Flavobacterium heparinum* or the cleavage of sialic acid by neuraminidases encoded by *Clostridium perfringens* and *Vibrio cholerae* (26–29). Bacteria also encode enzymes that can selectively cleave protein components of mucins coating the epithelial layer, promoting bacterial adherence and modulating numerous cellular pathways, as well as facilitating arboviral infection in mosquitoes (30–32). Many bacteria encode effectors and toxins that modulate cell signaling pathways to dysregulate cellular barrier complexes such as intercellular junctional proteins, including *Escherichia coli*, *Shigella flexneri*, *Helicobacter pylori*, *Salmonella* species, and *Vibrio* species (33–36). Under pathological conditions, such as bacterial sepsis, endotoxins produced from bacteria (including lipopolysaccharide) can also contribute to barrier dysfunction. The capacity to disrupt barriers is hypothesized to be beneficial for bacteria by promoting their adhesion to barrier cells, using components of the glycocalyx and extracellular matrix as a source of energy, and overall facilitating their dissemination throughout a host (30, 37, 38). In comparison to the field of virology, barrier modulation by bacteria is well studied, with many effectors and toxins characterized functionally and biochemically. In contrast, few viral factors directly modulating cellular barriers have been described.

## VIRAL TOXINS

While it is well appreciated that severe viral infections are associated with local and systemic vascular leak, the viral triggers of vascular leak are unclear, as is their impact on viral infection. A common hypothesis is that the leak is caused by a “cytokine storm,” resulting from high viral titers; however, the contributing viral and host factors are not well understood (39). Our work, and the work of others, over the past decade has implicated specific soluble viral proteins acting as “viral toxins” that trigger vascular leak via cell-intrinsic and cell-extrinsic mechanisms (40–44). We define this class of viral toxins as soluble or surface-exposed viral proteins that interact with endothelial and/or epithelial barrier cells to trigger signaling pathways that result in barrier dysfunction and vascular leak. This review summarizes our understanding of viral toxins from four distinct virus families with particular emphasis on flavivirus nonstructural protein 1 (NS1), Crimean-Congo hemorrhagic fever virus (CCHFV) glycoprotein 38 (GP38), SARS-CoV-2 Spike (S) protein, and Ebola virus (EBOV) glycoprotein (GP). We hypothesize that viral toxin-triggered barrier dysfunction is a common strategy utilized by viruses to disseminate within a host, thereby enhancing infection of target cells, simultaneously contributing to severe disease due to the pathological effects of both the virus and the induced vascular leak.

## FLAVIVIRUS NONSTRUCTURAL PROTEIN 1 (NS1)

Flaviviruses are members of the *Flaviviridae* family and contain mosquito- and tick-borne viruses, including human pathogens such as the four serotypes of dengue virus (DENV1-4), Zika virus (ZIKV), West Nile virus (WNV), Japanese encephalitis virus (JEV), yellow fever virus (YFV), and tick-borne encephalitis virus (TBEV). Flaviviruses pose a tremendous public health burden, with over half of the world’s population at risk of infection due to the habitat range of the vectors that transmit these viruses (45). Flavivirus disease burden is predicted to increase as rising global temperatures drive insect vectors into new habitats, expanding the number of people at risk of infection (46). The majority of flavivirus infections result in asymptomatic to “mild,” albeit debilitating, self-limiting febrile illnesses. In some cases, however, the disease can progress to severe, potentially fatal disease with distinct manifestations among flaviviruses (45, 47). For instance, severe dengue disease is mainly characterized by systemic vascular leak, resulting in hypovolemic shock that can lead to multiorgan failure and death (Fig. 1) (45, 47, 48). ZIKV infection can cause rare but severe disease manifestations, including microcephaly or fetal demise when pregnant mothers are infected (congenital Zika sSyndrome), as well as Guillain-Barré syndrome, a rare autoimmune disorder of the peripheral nervous system (45). Severe cases of West Nile and Japanese encephalitis are associated with encephalitis and neurological complications (45). Yellow fever virus infection can result in systemic manifestations, including vascular leak, with serious liver damage (45, 49). Clinically, predicting which patients will progress to severe disease is critical for case management, but the co-circulation of multiple flaviviruses in endemic areas, as well as undifferentiated febrile illnesses caused by other mosquito-borne viruses (e.g., chikungunya virus) or other microbial infections such as malaria and bacterial sepsis, complicates initial diagnosis and case management strategies in endemic areas.

Flaviviruses are enveloped viruses with a positive-sense RNA genome of ~11 kb encoding for three structural proteins and seven non-structural proteins, including NS1 (50). NS1 is critical for viral replication as a component of the flavivirus replication complex and also contributes to viral assembly and egress (51–55). Flavivirus NS1 is also actively secreted from infected cells, where it can associate with the cell surface as an obligate dimer or circulate in the extracellular space as a higher-order oligomer (hexamers and tetramers) with a lipid core (50, 56–59). Flavivirus NS1 has been used as a diagnostic antigen for decades, and the levels of NS1 in the sera of dengue and yellow fever patients have been found to correlate with disease severity and vascular leak (49, 60, 61). Although most literature refers to secreted DENV NS1, other flaviviruses also secrete NS1 (49, 62). Beyond its utility as a diagnostic antigen, the contribution of secreted NS1 to flavivirus infection and its contribution to flavivirus pathogenesis were unknown.

As early as the mid-1980s, it was demonstrated that vaccination with YFV NS1 or passive transfer of anti-YFV NS1 antibodies was protective against YFV challenge in primate and mouse models of infection (63, 64). Similar studies in a mouse model of DENV vascular leak syndrome showed protection against lethal challenge by NS1 vaccination and passive transfer of NS1-immune serum or anti-NS1 monoclonal antibodies (mAbs) (42). These observations led to the hypothesis that soluble flavivirus NS1 may function as a virulence factor contributing to severe disease manifestations, including vascular leak, during flavivirus infection (40–42). Over the past decade, increasing evidence has accumulated identifying distinct mechanisms by which secreted flavivirus NS1 proteins function as virulence factors by modulating the complement cascade, downregulating MHC molecules on immune cells, triggering inflammatory responses in immune cell populations, activating and depleting platelets, modulating PD1 signaling in T cells, inducing production of autoreactive antibodies, and triggering endothelial dysfunction through direct interactions with endothelial cells, all of which are proposed to contribute to severe dengue manifestations, including vascular leak (41, 42, 65–75). Flavivirus NS1 has also been shown to enhance virus transmission to mosquitoes during a blood meal and cause permeability in the midgut of *Aedes aegypti* (76, 77). Hence, soluble NS1 is referred to as a viral toxin, serving as a virulence factor independently of viral particles (40).

One ongoing question in the NS1 field is the relative contribution of NS1 interactions with immune cells versus endothelial cells to NS1-triggered vascular leak. NS1 interactions with immune cells have been documented to induce production of proinflammatory cytokines, which can contribute to the cytokine storm and vascular leak associated with severe dengue (41). These interactions with various immune cells, including human peripheral blood mononuclear cells (PBMCs), have been reported to be dependent on toll-like receptor 4 (TLR4) (41, 78), although this has been debated (79). Studies on endothelial dysfunction and vascular leak resulting from direct NS1 interactions with endothelial cells reveal that NS1 binds to heparan sulfate and chondroitin sulfate on the endothelial cell surface and is subsequently internalized via clathrin-mediated endocytosis, leading to the activation of enzymes that result in degradation of the endothelial glycocalyx layer (EGL) (Fig. 2). This disruption results in shedding of sialic acid, heparan sulfate, and chondroitin sulfate and mislocalization of intercellular junction components (49, 68–72, 80, 81). It is unclear what role TLR4-mediated activation of immune cells plays in DENV pathogenesis *in vivo* in mouse models since *TLR4*^-/-^ mice were equally susceptible to NS1-mediated vascular leak and lethal DENV challenge compared to *TLR4*^+/+^ littermate controls (69). Furthermore, recent work demonstrated that inflammasome activation, dependent on caspase 1/11, was important for protection and not pathogenesis in a murine lethal DENV challenge model (82). Collectively, these data highlight the complexity of the contribution of flavivirus NS1 to pathogenesis and immune protection and warrant further studies to define the roles of NS1 interactions with distinct cell populations.

Intriguingly, recent studies reported that multiple flavivirus NS1 proteins trigger endothelial dysfunction and vascular leak, but in a tissue-specific manner mirroring the disease tropism of their respective flavivirus (71, 72, 83) (Table 1). For example, DENV NS1 induces endothelial dysfunction and causes vascular leak in multiple tissues including the lung, brain, liver, and dermis, mirroring systemic disease tropism triggered by the virus. In contrast, WNV and JEV NS1 only interact with and trigger endothelial dysfunction in brain endothelial monolayers and cause vascular leak in the brain, mirroring the neurotropism of pathogenesis caused by these viruses. ZIKV NS1 induced endothelial hyperpermeability in both brain and umbilical vein endothelial cells as well as chorionic villi in placental explants, mirroring the pathogenesis of ZIKV infection in the brain and in fetuses, and YFV NS1 impacts the liver *in vitro* and *in vivo* most strongly, mirroring the liver tropism of YFV infections (45, 47, 71). These observations suggest that flavivirus NS1 may influence viral and disease tropism. Our recent work tests this hypothesis and demonstrates that indeed NS1 promotes virus dissemination across endothelial barriers, leading to increased viral load in distal tissues when NS1 was co-administered with virus and, conversely, lower viral load when anti-NS1 mAbs were co-administered with DENV infection (84). Furthermore, these studies revealed a putative homotypic interaction between virions and NS1, suggesting that both tissue-specific interactions between NS1 and endothelial cells as well as interactions between homotypic NS1 and virion pairs influence viral dissemination and tissue tropism of flaviviruses (84). These observations are substantiated by other studies demonstrating that ZIKV NS1 promotes dissemination of ZIKV across the blood-testis barrier, WNV and JEV NS1 promote virus dissemination across the blood-brain barrier, and DENV NS1 facilitates virus dissemination in mosquitoes (11, 77, 85, 86). Together, these data suggest that the capacity of flavivirus NS1 to trigger endothelial dysfunction and vascular leak may harbor an evolutionarily conserved benefit for viruses by allowing them to establish an infectious niche and disseminate throughout an organism. Indeed, encoding a vasoactive NS1 protein is highly conserved among circulating flaviviruses, and some highly virulent flavivirus isolates have enhanced capacity to secrete NS1 (87). Future studies are needed to more thoroughly evaluate this hypothesis.

**TABLE 1:** Summary of viral toxins with respective tissue and cellular tropism

Viral toxin	Viral family	Species	Endo/epithelial cell tropism *in vitro*	Vascular leak *in vivo*/*ex vivo*
NS1	*Flaviviridae*	DENV	Lung, brain, liver, dermis, umbilical vein endothelial cells	Lung, brain, liver, dermis
		ZIKV	Brain, umbilical vein endothelial cells	Brain, placental explants
		WNV, JEV	Brain endothelial cells	Brain
		YFV	Liver, lung endothelial cells	Liver, lungs
Spike	*Coronaviridae*	SARS-CoV-2	Lung endothelial and epithelial cells	Lung, spleen, small intestine
GP38	*Nairoviridae*	CCHFV	Liver, lung, and dermal endothelial cells	Liver and spleen
GP	*Filoviridae*	EBOV	Umbilical vein endothelial cells	n.d.^*a*^

^*a*^ n.d., not determined.

Importantly, the potential role of flavivirus NS1 in promoting virus dissemination and influencing flavivirus tropism suggests that soluble NS1 (and viral toxins in general) are underappreciated viral factors influencing virus tropism. Thus, understanding the molecular determinants and differential host factors required for flavivirus NS1 interaction with tissue-specific endothelial cells is essential. Numerous structural studies have revealed the unique structure of flavivirus NS1, identifying three distinct domains, namely, the β-roll, wing, and β-ladder (Fig. 3A) (56, 58, 88, 89). Recent studies have found that residues in the wing and β-ladder domains contribute to tissue-specific tropism of flavivirus NS1 *in vitro* and *in vivo* (90, 91). Specifically, these studies suggest that the wing domain of flavivirus NS1 contributes to initial cell binding of NS1 to glycans on the surface of endothelial cells, while the β- ladder mediates steps downstream of initial binding, including clathrin-mediated endocytosis and activation of intracellular enzymes such as cathepsin L and heparanase. Our current hypothesis is that differential interactions with glycans and host surface proteins dictate flavivirus NS1 cell and tissue tropism. Beyond this, NS1 has been shown to interact with components of high-density lipoproteins (HDL), including ApoA1, as well as scavenger receptor B1 and ephrin B1 (92–96). Differential interactions with lipid components in the blood may also impact tropism of flavivirus NS1, and differential levels of HDL species in patients may impact disease severity in infected individuals (87, 93).

**Figure:**
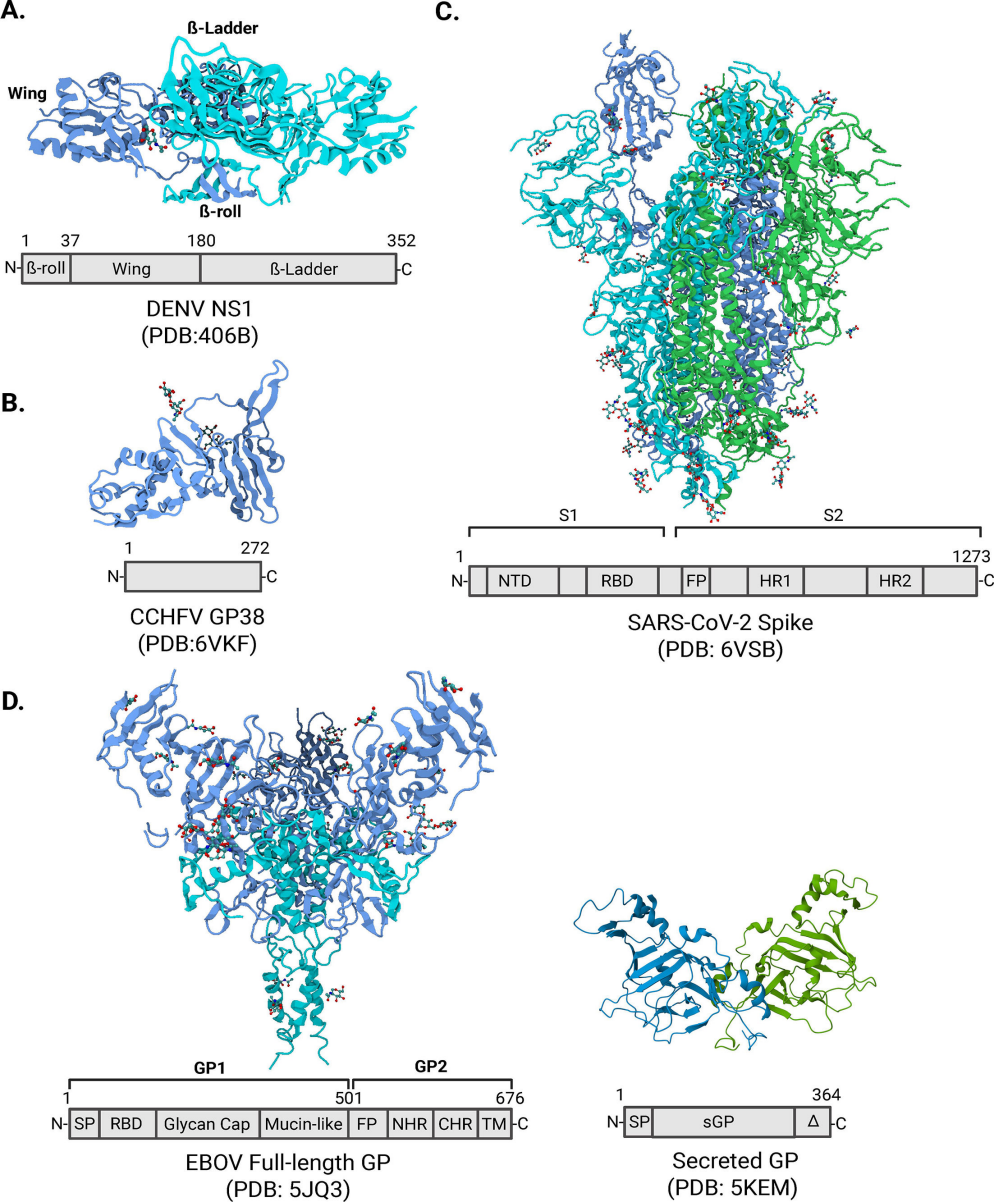
Fig 3 Structural details of viral toxins. Structures and linear domains of (**A**) DENV NS1 dimer (PDB 4O6B) (88), (**B**) CCHFV GP38 monomer (PDB 6VKF) (97), (**C**) SARS-CoV-2 Spike trimer (PDB 6VSB) (98), and (**D**) EBOV GP_1,2_ trimer (left, PDB 5JQ3) (99) and secreted GP (sGP) dimer (right) (PDB 5KEM) (100). Blue, turquoise, and green denote distinct monomers. Created in BioRender. (Biering Lab, 2026, https://BioRender.com/4w5u3bc).

Ultimately, the contribution of flavivirus NS1 to pathogenesis, viral dissemination, and tropism makes NS1 an attractive target for vaccine and therapeutic efforts. Indeed, passive transfer of anti-NS1 antibodies and vaccination against NS1 indicate that NS1 blockade is protective *in vivo*, lowering vascular leak, viral dissemination, and morbidity and mortality in mice (42, 63, 64, 84, 90, 101–106). Furthermore, small molecules targeting conserved features of NS1, including the capacity to interact with glycans or lipids, have also proven an effective strategy to alleviate flavivirus NS1-triggered pathogenesis (107–110). Additional studies are warranted to further develop these vaccines and therapeutic strategies. One factor that must be considered is to ensure that antibodies raised against flavivirus NS1 do not induce pathogenesis themselves. Previous studies suggested that some anti-NS1 antibodies may cross-react with antigens of the coagulation cascade or endothelial cells themselves, but the relative contribution of these cross-reactive events to DENV infection has not been demonstrated convincingly *in vivo* (67, 111–114). Furthermore, the relative contribution of NS1 to pathogenesis across diverse strains of DENV is unclear, as a recent study reported that regions in DENV prM/E, but not NS1, contributed to pathogenesis for a strain of DENV serotype 2 (115). More recent work using the same virus strain revealed a contribution of NS1 to DENV pathogenesis via modulation of PD1 expression in T cells (74). Nevertheless, strong evidence exists that targeting NS1 and considering NS1 as a component of future flavivirus vaccines is beneficial. While the biology underlying the role of flavivirus NS1 in promoting virus infection, dissemination, and pathogenesis is complex, it is clear that it plays multifunctional roles as a viral toxin.

## CRIMEAN-CONGO HEMORRHAGIC FEVER VIRUS GLYCOPROTEIN 38

CCHF is a widespread tick-borne disease present in Africa, Asia, and Europe (116). CCHFV infections lead to initial non-specific flu-like symptoms, which, in severe cases, can progress to vascular leak, extensive hemorrhage, multi-organ failure, and, in 10%–40% of cases, death. The virus encodes a negative- and ambi-sense, tri-segmented single-stranded RNA genome and belongs to the *Nairoviridae* family. The M segment encodes a polyprotein termed the glycoprotein precursor complex (GPC), which is proteolytically cleaved following translation (117). In addition to the nonstructural medium protein (NSm), the CCHFV M segment encodes two glycoproteins, Gn and Gc, which are derived from GPC cleavage products and cover the virion surface. Furthermore, the CCHFV GPC includes the GP85/160 polyprotein, which is cleaved into a mucin-like domain (MLD) and GP38 (118). Structurally, GP38 is composed of an N-terminal three-helix bundle, followed by a β-sandwich and a 12-amino acid disordered loop (Fig. 3B). Structural studies to date have not identified higher-order oligomers of GP38. GP38 has been shown to be involved in intracellular virus assembly and egress (119). Furthermore, GP38 is found in the supernatant of infected cells, likely due to its release from budding virions after proteolysis during egress (120, 121). Interestingly, analysis of serum and B cell responses in CCHF survivor cohorts showed that in addition to neutralizing antibodies targeting Gc, antibodies targeting GP38 were elicited (122, 123).

GP38 was first implicated as a potential virulence factor after a study demonstrated that a GP38-directed, non-neutralizing mAb protected immunocompromised mice from lethal CCHFV infections (124). The observed protection by this mAb (13G8) was shown to be independent of Fc gamma receptor (FcγR) binding (using LALAPG-modified mAbs) and signaling (using FcγR^−/−^ mice) but demonstrated a role for complement component C3 (97). Additionally, vaccines encoding GP38 alone or in combination with other viral antigens were shown to be protective in lethal CCHF mouse models (125, 126). The function of the extracellular form of GP38 was unclear, and the mode of action of the protective GP38-specific antibodies was not well understood.

In a recent publication from our group, GP38 was shown to cause hyperpermeability in several endothelial cell lines as well as vascular leak *in vivo* (Table 1) (44). Treatment of pulmonary and dermal endothelial cell monolayers resulted in a dose-dependent increase in hyperpermeability 6 h post-treatment, with kinetics similar to flavivirus NS1 (42). Importantly, concentrations of GP38 as low as 0.25 µg/mL were sufficient to trigger endothelial dysfunction, which is up to 40 times lower than the maximal concentration of GP38 circulating in mice during authentic CCHFV infection. Measurement of EGL components post-GP38 treatment revealed degradation of sialic acids and chondroitin sulfate, but, in contrast to flavivirus NS1, the amount of heparan sulfate on the endothelial cell surface did not significantly decrease (44). A possible explanation of these differences might be differential activation of enzymes that can degrade these EGL components (Fig. 2). Further differences from flavivirus NS1 may include a lack of endocytosis of GP38, indicating that the endothelial barrier dysfunction might be a result of intracellular signaling events triggered at the surface of the endothelial cells.

Administration of recombinant GP38 in the dorsal dermis of mice led to increased extravasation of tracer dyes from the bloodstream into the dermis, indicating induction of vascular leak. Similar levels of dextran extravasation after injection of 5 µg of GP38 and 15 µg of DENV NS1 were observed, supporting the higher potency of GP38 observed *in vitro*. When GP38 was administered systemically to mice via intravenous injection, a significantly increased amount of Evans Blue was measured in the liver and spleen, indicating vascular leak. Histological analysis confirmed signs of endothelial dysfunction, including edema, collagen dispersal, and congested veins. Additionally, when exogenous GP38 was co-administered during CCHFV infection in a mouse model, it further enhanced vascular leak and viral dissemination into the liver, spleen, and kidney, physiologically relevant organs in relation to human disease. Finally, antibodies targeting GP38 were able to decrease hyperpermeability *in vitro* and reduce GP38 levels in serum, vascular leak in the liver, and viral load in distal tissues. These data indicate that GP38 promotes viral dissemination of CCHFV, playing a similar role as flavivirus NS1 (44).

In summary, current evidence suggests that CCHFV GP38 can act as a viral toxin that disrupts endothelial barrier function, thereby enhancing vascular leak and viral dissemination. It represents an attractive target for pharmacological and antibody-based interventions and should be considered in vaccine approaches. Importantly, recent advances in the structure-based design of CCHFV glycoproteins enabled the generation of a construct encoding a stabilized multimer of CCHFV Gc, Gn, and GP38 as a potential immunogen (121).

## CORONAVIRUS SPIKE (S)

The *Coronaviridae* encompass a family of respiratory viruses containing human pathogens that can cause mild respiratory illnesses, including human coronaviruses (HCoV) 229E, OC43, and NL63 that cause the common cold (127). This family also includes recently emerged pathogens that cause severe, potentially fatal, acute respiratory distress syndrome (ARDS), including the severe acute respiratory syndrome coronavirus (SARS-CoV-1, formerly SARS-CoV), Middle Eastern respiratory syndrome coronavirus (MERS-CoV), and SARS-CoV-2, the causative agent of the COVID-19 pandemic, which has had a devastating impact on society globally (128). Severe COVID-19 is associated with vascular leak in the lungs characterized by pulmonary edema, contributing to ARDS (128–131). SARS-CoV-2 is also associated with pathology in extra-pulmonary organs, including the intestine and brain, and displays expanded tropism compared to SARS-CoV-1 and MERS-CoV, although this is confounded by the fact that there are several orders of magnitude more COVID-19 cases available for study compared to SARS or MERS cases (Fig. 1) (132). While vascular leak was accepted to be a hallmark of severe COVID-19, the viral triggers and host factors contributing to this pathogenesis were unknown and generally explained as a “cytokine storm” resulting from uncontrolled viral infection.

The primary surface glycoprotein of coronaviruses mediating cell entry is Spike (S), which is comprised of two domains—S1 containing the receptor-binding domain (RBD) and S2 harboring the membrane fusion machinery (Fig. 3C) (128). S from SARS-CoV-2 and SARS-CoV-1 interacts with angiotensin-converting enzyme 2 (ACE2) on the surface of cells to trigger internalization and cell-virus fusion (133–136). Previous work suggested that interactions between SARS-CoV-1 S and ACE2 result in a dysregulation of the renin-angiotensin pathway, contributing to ARDS and inflammation associated with SARS (137). We investigated this hypothesis for SARS-CoV-2 S, resulting in the discovery that SARS-CoV-2 S functions as a vasoactive viral toxin that promotes endothelial and epithelial barrier dysfunction *in vitro* and vascular leak *in vivo* (43). Surprisingly, this phenotype was independent of ACE2 interactions but instead dependent on heparan sulfate and integrins on the cell surface (43). Previous work demonstrated an enhanced capacity of SARS-CoV-2 S to interact with heparan sulfate, compared to SARS-CoV-1 S, due to the evolution of positively charged amino acids present in the RBD of SARS-CoV-2 S (138). We found that SARS-CoV-2 S first binds to heparan sulfate on the cell surface before engaging integrins, resulting in the release of transforming growth factor beta (TGFβ) from the cell surface, which in turn interacts with TGFβ receptor (TGFBR), an event required for S-triggered barrier dysfunction (43). Downstream of TGFBR signaling, enzymes including heparanase, matrix metalloproteinase 9, and A disintegrin and metalloprotease 17 (ADAM17) are required for modulation of the EGL and intercellular junctional complexes (Fig. 2) (43).

While we established SARS-CoV-2 S as a vasoactive viral toxin, many outstanding questions remain. First, what benefit, if any, does S-mediated vascular leak have for virus infection? We hypothesize that the capacity of SARS-CoV-2 S to trigger vascular leak in the lung can promote dissemination of virus out of the lung and into secondary sites of infection, potentially contributing to extra-pulmonary pathology and the broad tissue tropism of SARS-CoV-2. An additional benefit may be making epithelial and endothelial barrier cells more sensitive to SARS-CoV-2 infection. Our previous work demonstrated that membrane-tethered mucins (specifically MUC1 and MUC4) restricted SARS-CoV-2 cell attachment and infection of cells *in vitro* and *in vivo* (139). As cellular pathways triggered by SARS-CoV-2 S facilitate shedding of diverse components of the EGL, this infection-independent pathway presumably acts to thin out the EGL in the lung and endothelium and enhance SARS-CoV-2 infection of barrier cells by decreasing glycan-mediated steric hindrance. While *in vivo* and *in vitro* evidence exists supporting this hypothesis, it is still unclear if other signaling pathways triggered by S may modulate viral infection (140, 141). Other outstanding questions include whether differences in cellular interactions of S dictate tissue tropism of coronaviruses and the capacity to trigger inflammation within a tissue. Furthermore, the relative contribution of anti-S antibodies to blocking viral infection (inhibition of S-ACE2 interactions) versus blocking S-mediated barrier dysfunction (inhibition of S-glycan and S-integrin interactions) to protection against severe COVID-19 should be investigated.

## EBOLA VIRUS GLYCOPROTEIN (GP)

Ebola virus (EBOV) belongs to the *Filoviridae* family and contains a negative-sense RNA genome (142). EBOV was first discovered in the Democratic Republic of the Congo in 1976, and 6 distinct species have been identified in Africa since then (143). EBOV is thought to be transmitted to people from bats and non-human primates or through human-to-human transmission during direct contact with bodily fluids of infected patients (144). EBOV infection is deadly in humans, with a case fatality rate ranging from 30% to 90%, and is characterized in severe cases by vascular leak, hypotension, shock, coagulation disorders, hemorrhage, and multiorgan failure (Fig. 1) (144–146). Interestingly, the underlying vascular pathologies overlap with the other diseases described above (146). Severe disease driven by EBOV infection is generally characterized as a cytokine storm, with triggers of pathology attributed to proinflammatory mediators (including TNF-α, IL-1β, and IL-6), infection-triggered cell death, and potentially viral proteins themselves (147, 148). Like the viruses discussed above, the relative contribution of these factors to viral pathogenesis is not clear.

The EBOV GP coats the surface of the virion and mediates viral entry via interactions with the host receptor Niemann-Pick C1 (NPC1) in the late endosome, which facilitates membrane fusion (149–152). Beyond viral entry, EBOV GP plays a role in immune evasion and pathogenesis. The EBOV GP gene encodes two distinct glycoproteins resulting from transcriptional editing (153). The glycoprotein coating the virion is a trimeric class I fusion protein denoted GP_1,2_ that mediates viral entry (Fig. 3D) (154). The membrane-bound GP_1,2_ trimer can also be shed from the surface of infected cells following cleavage by ADAM17, leading to shedding of the soluble GP_1,2_ ectodomain (155). Shed GP_1,2_ (shed GP) is believed to play a role in immune evasion and pathogenesis by serving as an antibody decoy as well as triggering production of pro- and anti-inflammatory cytokines (TNF-α, IL-1β, IL-6, IL-8, IL-12p40, IL-1RA, and IL-10) (156). The trimeric GP_1,2_ is the minor form of EBOV GP, with the major form being the soluble EBOV GP dimer (sGP) that is secreted during infection (Fig. 3D). High levels of sGP have been reported in the serum of EBOV-infected patients, non-human primates, and rodents, and it has been hypothesized to also function as an antibody decoy (154, 157–159). Interestingly, EBOV mutants that do not produce sGP mutate to regain sGP expression *in vivo*, suggesting an important role of sGP during infection (160, 161). Recent studies suggest that sGP facilitates virus infection by mediating an increase in virus uptake into late endosomes as well as promoting virus replication through modulation of the mitogen-activated protein kinase (MAPK) signaling pathway. In mice, administration of sGP following EBOV infection leads to increased viral titers in the liver and spleen (162).

Membrane-bound GP_1,2_, shed GP, and sGP have been shown to promote endothelial dysfunction and vascular leak through interaction with and activation of endothelial and immune cells (147, 156, 163–166). Intriguingly, the capacity of shed GP to activate endothelial and immune cells has been reported to correlate with virulence in humans, as EBOV Reston shed GP, which is non-pathogenic in humans, does not trigger endothelial dysfunction, while the pathogenic EBOV Zaire shed GP does (165, 166). The role of sGP in pathogenesis is less clear, as work suggests that sGP may act to diminish inflammatory responses in uninfected cells, thus potentially limiting antiviral inflammatory responses, proposed to promote viral dissemination (167). Others have reported that sGP does not trigger endothelial dysfunction and, in fact, may play a vasoprotective role in some contexts (147). This discrepancy could be explained by differences in cell types used as well as overall methodology, and further studies are needed to investigate how diverse tissue-specific endothelial cells respond to EBOV shed GP and sGP *in vitro* and *in vivo*. Beyond membrane-bound GP_1,2_, shed GP, and sGP, recent studies found that cleavage of the EBOV sGP protein by the host furin protease results in the production and secretion of a small protein called delta peptide (∆ peptide) (168). The ∆ peptide was shown to act as an enterotoxin, leading to intestinal villus destruction, as well as vacuolization and thinning of the basement membrane of the small intestine, contributing to EBOV-induced severe diarrhea and revealing an additional mechanism by which EBOV GP contributes to pathogenesis beyond viral entry (168).

Altogether, these observations, especially those regarding the shed and secreted GP products, fit the definition of viral toxins discussed above. In light of these studies, a plausible hypothesis is that secreted or shed GP are able to circulate during infection and interact with barrier and immune cell populations to activate signaling pathways that promote the EBOV virus lifecycle and facilitate dissemination, contributing to disease severity. Further work is required to test these hypotheses, as well as to compare the capacity of EBOV shed GP and sGP from different virus species to trigger vascular leak. Defining the relative contribution of EBOV membrane-bound GP, shed GP, sGP, and the ∆ peptide in EBOV-triggered endothelial dysfunction and vascular leak is critical, as is evaluating the therapeutic potential of targeting this pathway during infection.

## CONCLUSION

The aim of this minireview is to advance a unifying hypothesis attempting to explain why viruses encode viral toxins and trigger vascular leak. We base this hypothesis on examples in the flavivirus, coronavirus, bunyavirus, and filovirus literature. Based on our recent work and existing literature, we hypothesize that the capacity to disrupt endothelial and epithelial barriers benefits the viral lifecycle by facilitating infection of target cells within a primary site of infection and promoting dissemination across barriers, allowing viruses to gain access to distal secondary sites of infection. We propose that this promotes virus replication in target cells to achieve high viral titers required for transmission of some viruses and leads to increased pathogenesis. We define this class of viral toxins as either secreted or surface-exposed viral proteins that possess vasoactive or inflammatory properties, modulating endothelial and/or epithelial barriers to promote viral dissemination. We hypothesize that this is a common mechanism and potentially an example of convergent evolution amongst many viral families. Furthermore, and perhaps most critically, our model predicts that therapeutically targeting these viral proteins or host pathways triggered by these viral proteins is a novel strategy to inhibit virus pathogenesis. Anti-viral toxin or anti-leak therapeutics may serve as virus-agnostic therapeutic strategies for treating many viral infections.

We acknowledge that the notation of viral toxin for this new class of viral proteins requires some discussion. While the vasoactive properties (or capacity to trigger vascular leak) of our four examples are the common theme discussed in this article, we want to emphasize that each of these viral proteins has numerous reported functions that may contribute to overall pathogenesis, including triggering production of proinflammatory cytokines from immune cells, acting as antibody decoys, modulating the complement cascade, downregulating activity of specific immune cell populations, perturbing the blood clotting cascade, and others briefly touched on above. Given their diverse roles in modulating the biology of uninfected cells, we believe that viral toxin is an appropriate designation. We also considered the blanket term virulence factor, but this does not differentiate between viral proteins that are directly tied to virus infection from virulence factors acting on uninfected cells. Furthermore, the activities of viral toxins have parallels with bacterial exotoxins in that they modulate barriers and perturb cellular pathways in a manner that benefits the microbe (virus or bacteria) but can act on uninfected cells to cause pathogenesis. In general, bacterial toxins seem to be overall more cytotoxic than the vasoactive class of viral toxins described here.

On the topic of cytotoxicity, we want to clarify that we propose the term viral toxin be defined as any viral protein that contributes to pathogenesis by acting on uninfected cells, not only the vasoactive proteins discussed in detail in this perspective. For example, the classically described and prototypical viral toxin, rotavirus NSP4, certainly fits this definition as well (169–171). Rotavirus NSP4 is a viroporin, or viral protein possessing the capacity to form pores in cellular membranes, that triggers Ca^2+^ and other ion efflux from cells and contributes to diarrhea during rotavirus infection (172, 173). However, a major distinction between rotavirus NSP4, as with other characterized viroporins, and vasoactive viral toxins is that the latter do not appear to form pores in cell membranes nor trigger overt cellular cytotoxicity on their own. Thus, rotavirus NSP4 may function more similarly to bacterial exotoxins (forms cytotoxic pores). We propose that there may be distinct types of viral toxins, with vasoactive viral toxins that promote dissemination being one such subclass.

There are a few caveats to our overall hypothesis that are important to consider. First, the role of viral toxins may have distinct contributions to infection versus pathogenesis early during infection compared to late in infection. Our data suggest that viral toxin promotion of viral infection via the removal of glycan steric hindrance and crossing of barriers to promote viral dissemination is a key event early during infection. These early events likely result in local endothelial dysfunction at both primary and secondary sites of infection, but not the overt, systemic vascular leak associated with severe viral infections. Late during infection, the impact of viral toxins on barrier cells and immune cells may be more overtly pathogenic, contributing—alongside viral infection—to the multifactorial cytokine storm associated with severe disease manifestations. At these later time points, it is challenging to differentiate the role of viral toxins in disease state, and the multifactorial nature of late-stage pathology may mean that therapeutic treatments blocking viral toxins directly or host pathways activated by viral toxins may be most effective earlier during infection. Furthermore, we find that the role of viral toxins in promoting viral dissemination, vascular leak, and overall pathogenesis is dependent on the initial dose of virus used to infect mice. If the virus dose is above a certain threshold, the initial inflammation triggered by the virus, regardless of the presence or absence of viral toxin, is sufficient to trigger vascular leak and promote viral dissemination. This seems to be the case for some highly pathogenic strains of DENV in Southeast Asia (115). Of course, the role of a viral toxin in promoting viral dissemination and contributing to pathogenesis will be highly dependent upon the specific virus and may differ across distinct sites of infection. We also acknowledge that the hypothesis that viral toxins are an example of convergent evolution promoting viral dissemination into distinct tissues is less clear when considering neurotropic viruses, where invasion of the brain does not possess a clear evolutionary benefit. For many neurotropic viruses, neurovirulence is likely an evolutionary “dead-end” with potentially no benefit to virus infection, serving more as “collateral damage.”

Finally, the potential of therapeutics targeting viral toxins, or the cellular pathways they activate, to alleviate pathogenesis is promising. Knowing how to target these viral proteins necessitates further research to gain a deeper understanding of their mechanisms of action. It is unclear if particular viral toxins require unique or conserved host cell pathways to trigger endothelial dysfunction or immune cell interactions. Our early investigations into the mechanisms of action of flavivirus NS1 and SARS-CoV-2 S suggest that differential host factors on the cell surface, including glycan attachment factors and proteinaceous cellular receptors, may be unique across viral toxins, as well as unique among viral toxins within a virus family. This is apparent for flavivirus NS1, which seems to have differential cell binding properties that closely mirror tropism of a given virus. The amino acids driving these tissue-specific differences are highly charged and may contribute to differential glycan and receptor interactions (71, 91). Downstream of initial interactions of a viral toxin with a cell, the activation of key cellular enzymes and kinases appears to be mostly conserved, as a downstream consequence of viral toxin signaling on cells is the activation of enzymes and kinases that result in the breakdown of the EGL and intercellular junction complex. Nonetheless, there seems to be some specificity in these downstream signaling pathways. First, flavivirus NS1 requires cathepsin L to trigger endothelial dysfunction, while cathepsin L is dispensable for SARS-CoV-2 S-triggered barrier dysfunction (43, 68, 69). Furthermore, CCHFV GP38 appears not to be able to induce disruption of heparan sulfate *in vitro*, in contrast to flavivirus NS1 and SARS-CoV-2 S (43, 44, 68). Understanding if differences in the tissue tropism, pathogenesis, or initial site of infection of a given virus dictate differences among viral toxins will be important to address. For example, viruses that enter at mucosal surfaces like SARS-CoV-2 versus viruses that enter in the skin and proceed directly into the bloodstream like flaviviruses and CCHFV versus filoviruses that enter humans through broken skin or mucosal surfaces may utilize their viral toxins in different manners. Furthermore, do viral toxins of viruses that trigger hemorrhagic fever (breakdown and cell death of endothelial cell barriers) function distinctly from viruses that do not display hemorrhagic manifestations? Perhaps they are more cytotoxic or activate more inflammatory pathways, or simply different pathways, in endothelial cells? The answers to these questions will dictate whether therapeutics can be developed to target all viral toxins in a virus-agnostic manner or if more targeted approaches must be taken.

In summary, this review serves to unify virological and clinical observations from disparate virus families and proposes that mechanisms of pathogenesis long studied in isolation may have a common mechanistic route. We propose that viral toxins are an example of convergent evolution among numerous viral families and possess the conserved functions of disrupting physiological barriers to promote infection at primary sites and dissemination to secondary sites of infection. As a new field, research efforts should be prioritized to (i) define novel viral toxins, (ii) define the mechanisms of action of currently identified viral toxins, (iii) differentiate the role of viral toxins in modulating distinct cell types (most critically immune vs. barrier cells), and (iv) determine how viral toxins define tropism of virus infection. The discovery of viral toxins has the potential to provide a new basis to target viral pathogenesis agnostically, which is critical today more than ever given the large number of emerging and re-emerging viral infections. The translation of basic science investigating viral toxins has the potential to produce a new arsenal of anti-leak therapeutics that can be used to treat viral infections long before specific vaccines can be produced.
